# Supplementary material for: Chromothripsis during telomere crisis is independent of NHEJ, and consistent with a replicative origin
Source: Genome Res. 2019 May;29(5):737–49. doi: 10.1101/gr.240705.118 (PMC6499312; doi:10.1101/gr.240705.118)
Supplement: Supplemental Material [file supp_gr.240705.118_Supplemental_file_1.zip › contigs/annotated_contigs/DB77/contig.2.DB77_length_352_mean_cov_8.52272727273.docx]

**DB77_length_352_mean_cov_8.52272727273**

```
AATGCTGCCTCTATGAGTCTGTGCACCAGTACATGGATGCATGGTATGACACAACACATGACAAGAATGAGTACACCTACTGCAGCTGC
 >chr8:5680603-5680798 + E=6e-88
GAGACAAATAAGACTTGAGGCTATGATTCCTTTCCATTTACCGAACCATTTTTCTAGCCACCTTGTAAAGGGATCATTTACCCTTGAGT

TTTTGGCTAACTCATTG|T|ACACAGCTCTGGGCTGGGTACCTGGAAGCTCACGAGCTTGTTTGGCCTCCCCTCCTGGACACAGGAGGT
                   >chr8:952504-952660 + E=2e-82
GTCAAGGCTGTGGTCAGCAGAATCTACCCAGCTGGACACTGAGTGGACGCCCTGTGGGGTGGCTGTGAAATGGGTCCACTTTGTAGT
```
